# Resolving the Speciation Patterns and Evolutionary History of the Intercontinental Disjunct Genus *Corylus* (Betulaceae) Using Genome-Wide SNPs

**DOI:** 10.3389/fpls.2018.01386

**Published:** 2018-10-25

**Authors:** Zhen Yang, Tian-Tian Zhao, Qing-Hua Ma, Li-Song Liang, Gui-Xi Wang

**Affiliations:** Key Laboratory of Tree Breeding and Cultivation of the State Forestry and Grassland Administration, Research Institute of Forestry, Chinese Academy of Forestry, Beijing, China

**Keywords:** *Corylus*, speciation, recombination, hybridization, divergence time estimation, ancestral area reconstruction, genome-wide SNPs

## Abstract

Understanding the underlying mechanisms of species origin, divergence, and distribution patterns of the intercontinental disjunct taxa has long fascinated botanists. Based on 4,894 genome-wide single-nucleotide polymorphism dataset, we present a molecular phylogenetic reconstruction of genus *Corylus* (Betulaceae), which have a disjunct distribution between Eurasia and North America (NA). The aim is to explore the speciation patterns and evolutionary relationships of *Corylus* species by establishing a general phylogenetic framework with extensive sampling. Both the molecular phylogeny inferred from recombination-free dataset and structure analysis support the division of *Corylus* into four major clades (A–D). Recombination tests and hybridization detection reveal extensive recombination and hybridization events among different clades, which have potentially influenced the speciation process of *Corylus*. Divergence time estimation indicates that recent common ancestor (MRCA) of *Corylus* occurred in late Eocene (∼36.38 Ma) and subsequent rapid diversification began during Miocene. Ancestral area reconstruction shows that *Corylus* originated from southwest China. The arrival of two clades (Clades B and C) to NA was well supported by the long distance dispersal crossing the Bering land bridge. The Himalayas, European-Mediterranean area, and other distribution regions are primarily the recipients of dispersal taxa. Vicariance after dispersal plays an important role in speciation.

## Introduction

In the Northern Hemisphere, intercontinental disjunction of related plant species among eastern Asia (EA), Europe, and North America (NA) has long fascinated botanists and biogeographers (Tiffney and Manchester, 2001; Xiang and Soltis, 2001; Donoghue and Smith, 2004; Wen and Ickert-bond, 2009; Xiang et al., 2015). The disjunction patterns have been utilized to grasp the histories of plant dispersal between continents as well as allopatric speciation (Boufford and Spongberg, 1983; Wen, 1999; Kelchner and Bamboo Phylogeny Group, 2013). Multiple origins and complex evolutionary patterns of this intercontinental disjunction have been discussed based on fossils, molecular, and geologic evidence (Wen, 1999; Xiang et al., 2004). Despite various interpretations for the disjunction based on different taxa, it has been generally recognized that climatic fluctuations over the Cenozoic and two intercontinental land bridges, i.e., the North Atlantic Land Bridge (NALB) and the Bering land bridge (BLB), have played important roles in shaping current disjunctions of the Northern Hemisphere flora. Nevertheless, due to the complex biotic responses to diverse abiotic factors in the Northern Hemisphere, there still remains much to explore about the evolutionary history of the disjunction patterns and the underlying mechanisms of species diversification.

Understanding the speciation of diverse lineages or species-rich communities is of great interest in biological sciences. The speciation mechanisms may be associated with various factors such as probable gene mutations, potential recombination, and hybridization phenomena, and a series of dispersal and vicariance events. Mutation and gene recombination provide the original impetus for biological evolution, which is the intrinsic factor of speciation, while selection will externally retain dominant species that can be well suitable for certain ecological conditions. Especially, closely related species may also hybridize if reproductive isolation is incomplete, leading to diverse possible outcomes such as the decline or extinction of one or both parental species through genetic or demographic swamping (Holt and Gomulkiewicz, 1997; Wolf et al., 2001), establishment of recombinant species (Livingstone and Rieseberg, 2004), or the transfer of adaptive alleles (Arnold et al., 2016). Furthermore, more studies indicate that speciation has resulted from dispersal or a complex mix involving both vicariance and dispersal (Mao et al., 2010; Migliore et al., 2012; Chen et al., 2014). However, comprehensive analyses including all these aspects are very few.

*Corylus* L. (Betulaceae), the hazelnut genus, provides an ideal model for studying the evolution of intercontinental disjunctions in the Northern Hemisphere, as well as the diversification within EA. The genus consists of approximately 15–20 species disjunctly distributed in major areas of the Northern Hemisphere, with high species diversity in EA (especially in China). About 10 species occur in China, 1 in Korea and Japan, 3 in NA, 1 in the Himalayas, and 2 in Europe and the Mediterranean regions. Although the distribution of *Corylus* species shows a noticeable disjunct pattern, the biogeographical study by Whitcher and Wen (2001) is the only study that examined this pattern by calculating the substitution rate of the internal transcribed spacer (ITS) region. Additionally, age estimation of *Corylus* has not been conducted in spite of abundant fossil records (Crane, 1989; Chen et al., 1999). Therefore, origin of genus *Corylus* species and their biogeographic patterns have not been addressed.

The genus *Corylus* is characterized by several morphological synapomorphies, including large animal-dispersed nuts, hypogeal seed germination, and filaments that are completely divided longitudinally (Chen et al., 1999). The chromosome number of this genus is 2*n* = 2*x* = 22 (Thompson et al., 1996). Classification in the genus has traditionally been based on morphology, especially in the husk or involucre (Krüssmann, 1976; Everett, 1982; Huxley and Griffiths, 1999). The number of *Corylus* species has varied depending on various authors. Infrageneric taxonomy has recognized two main sections or subgenera (*Acanthochlamys* and *Corylus*); with section *Corylus* often being divided into three subsections (Furlow, 1997). While several classification treatments have been limited to taxa in a regional scale (Li and Cheng, 1979; Liang and Zhang, 1988; Furlow, 1997), even among classifications treating the same species, inclusion of taxa within each section or subgenus has varied significantly. Species identification is also controversial in *Corylus*. Particularly, two species complexes have been subjected to different taxonomic interpretations: *Corylus heterophylla* Fisch. complex and the *C.* *cornuta* Marsh. complex (Li and Cheng, 1979). The *C. heterophylla* complex distributes widely in EA, including three leafy-husked shrubs: *C. heterophylla* Fisch, *C. kweichowensis* Hu, and *C. yunnanensis* Fisch. The *C. cornuta* complex is a group of EA and NA taxa which contain four bristle-husked shrubs: *C. cornuta* Marshall, *C. californica* Marshall, *C. sieboldiana* Blume, and *C. mandshurica* Maxim. The species within each complex have been variously lumped and split. Over the past decades, several molecular studies have provided important insights into the phylogeny and taxonomy of *Corylus* (Erdogan and Mehlenbacher, 2000; Whitcher and Wen, 2001; Bassil et al., 2013). However, these above studies are still failed to reach an ideal result partly because of the incomplete taxa sampling and partly due to the low resolution in species delimitation.

In this study, we sampled extensively from across the known geographical range of *Corylus* and performed multiple analyses based on the genome-wide single-nucleotide polymorphism (SNP) dataset. Our aims are (1) to establish a robust molecular phylogeny and reveal the evolutionary relationships of *Corylus*; (2) to test the potential recombination and hybridization events that might affect the speciation of *Corylus*; and (3) to estimate the divergence time and history biogeography of *Corylus.*

## Materials and Methods

### Study System

**FIGURE 1:**
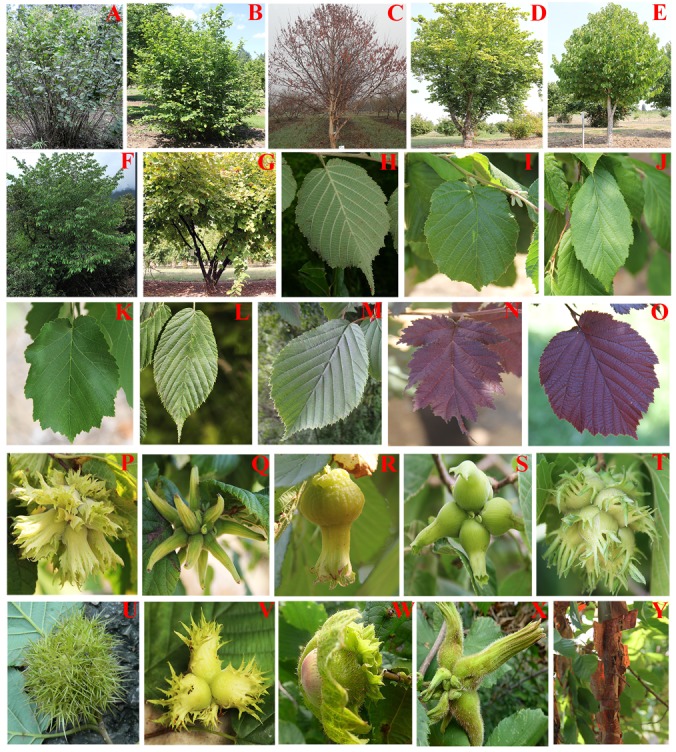
Phenotypic characters of several representative *Corylus* species. **(A–G)** Tree forms. **(H–O)** Leave shapes. **(P–V)** Husk shapes. **(W,X)** Husk hairs. **(Y)** Stem.

Members of genus *Corylus* are perennial shrubs or trees that vary most notably in the tree trunk, leaf, and involucre characteristics (Figure 1). Approximately 16–20 species occur across Eurasia and NA, with East Asia especially China as the main species enrichment zone. We employed the accepted names from *The Plant List*^1^ and *Flora of China* (Editorial Board of the Flora of China of Chinese Academy of Sciences, 2013), and replenished it by referring to other relevant floristic treatments: *Flora Europaea* (Tutin and Walters, 1993), *Flora of NA* (Furlow, 1997), *Flora of the USSR* (Bobrov, 1936), and *Flora of Japan* (Ohwi, 1965). Simultaneously, we consulted the herbarium specimens of Institute of Botany, Chinese Academy of Sciences. For further correction, we compared the existing identification with that of *World Checklist of Selected Plant Families* (WCSP) so as to filter synonymy or unresolved species names. Here, several scenarios for the treatments of ambiguous species were described. (1) Despite of an accepted name in *The Plant List, C. colchica* has few records (Bobrov, 1936; Govaerts and Frodin, 1998) and remains a bit of uncertainty (Holstein et al., 2018). Therefore, this alleged species is not considered to be included in our samples. (2) *C. maxima*, one of the most mysterious species, has been widely reported as a wild species occurring in European-Mediterranean area (Bobrov, 1936; Tutin and Walters, 1993), however, relevant phylogenetic analysis (Whitcher and Wen, 2001) has revealed that it is probably a variety of *C. avellana.* Thus, *C. maxima* was covered and represented by *C. avellana* in this study. (3) Although the two Chinese species *C. potaninii* (Govaerts and Frodin, 1998) and *C. wulingensis* (Govaerts and Frodin, 1998) are both designated as accepted names in *The Plant List*, they are no longer recognized as distinct *Corylus* species and not mentioned in *Flora of China* (Editorial Board of the Flora of China of Chinese Academy of Sciences, 2013). Recent classification in China has confirmed them as the same species with the name *C. kweichowensis* (Liang and Zhang, 1988; Editorial Board of the Flora of China of Chinese Academy of Sciences, 2013). (4) As for the synonym, *C. mandshurica* and *C. californica* are separately regarded as the variety of *C. sieboldiana* var. *C. mandshurica* (Ohwi, 1965) and *C. cornuta* subsp. *californica* (Furlow, 1997) by some taxonomists, but as distinct species by others (Bassil et al., 2013). In view of the significant geographical isolation and covering more taxa, we treat them as four distinct species so as to conduct a comprehensive species analysis.

### Taxon Sampling and DNA Extraction

Based on the above pre-treatments, we finally chose as inclusive as possible the 17 taxa which represented the most complete examination of the genus *Corylus* to date. In fact, our team has initiated a comprehensive investigation and collection for *Corylus* germplasm resources since 2010 by cooperating with relevant research institutes in China, America, Turkey, and Netherlands. Therefore, we are convenient to collect all the 17 extant *Corylus* taxa, and the species delimitation work received great support from these specialists. According to our survey, nearly all the *Corylus* resources are under wild or semi-wild state except for *C. avellana* which has numerous of cultivars in the market. Consequently, various nature nurseries were *in situ* established by local forestry institutions to protect these wild resources without disrupting their genetic diversity. Ten (eight species and two varieties) of the 17 taxa were taken from the natural populations that covered their distribution ranges in China. The remaining seven taxa were friendly provided by the herbarium specimens deposited in countries and regions of Europe, NA, and the Himalayas. It is worth noting that these specimens were also collected from the representative natural populations across their distribution areas (Table 1). Overall, a total of 45 specimens were collected, of which, the ingroup included 42 samples representing 17 *Corylus* species (varieties), while the other three samples of *Ostryopsis davidiana* Decne were chosen as the outgroup. Voucher specimens were deposited in the Economic forest research office of Research Institute of Forestry Chinese Academy of Forestry, Beijing, China.

**Table 1:** Details of taxon code, sample code, and sampling location of 45 individuals used in the study.

No.	Taxon code	Sample code	Location
1	Taxa 1	*C. colurna-1*	Poland
2		*C. colurna-2*	Czech Republic
3		*C. colurna-3*	Georgia
4	Taxa 2	*C. jacquemontii-1*	Pakistan
5		*C. jacquemontii-2*	Pakistan
6		*C. jacquemontii-3*	Nepal
7	Taxa 3	*C. fargesii-1*	Gansu, China
8		*C. fargesii-2*	Gansu, China
9	Taxa 4	*C. chinensis-1*	Yunnan, China
10		*C. chinensis-2*	Shanxi, China
11		*C. chinensis-3*	Shanxi, China
12	Taxa 5	*C. avellana-1*	Oregon, United States
13		*C. avellana-2*	Oregon, United States
14		*C. avellana-3*	Oregon, United States
15	Taxa 6	*C. americana-1*	Michigan, United States
16		*C. americana-2*	Oregon, United States
17		*C. americana-3*	Oregon, United States
18	Taxa 7	*C. yunnanensis-1*	Yunnan, China
19		*C. yunnanensis-2*	Yunnan, China
20		*C. yunnanensis-3*	Yunnan, China
21	Taxa 8	*C. kweichowensis-1*	Shanxi, China
22		*C. kweichowensis-2*	Shanxi, China
23	Taxa 9	*C. kweichowensis* var. brevipes	Jiangxi, China
24	Taxa 10	*C. heterophylla-1*	Hebei, China
25		*C. heterophylla-2*	Jilin, China
26	Taxa 11	*C. sieboldiana-1*	Japan
27		*C. sieboldiana-2*	Japan
28		*C. sieboldiana-3*	Korea
29	Taxa 12	*C. mandshurica-1*	Beijing, China
30		*C. mandshurica-2*	Hebei, China
31	Taxa 13	*C. californica-1*	Oregon, United States
32		*C. californica-2*	Oregon, United States
33		*C. californica-3*	Oregon, United States
34	Taxa 14	*C. cornuta-1*	Minnesota, United States
35		*C. cornuta-2*	Minnesota, United States
36		*C. cornuta-3*	New York, United States
37	Taxa 15	*C. ferox-1*	Yunnan, China
38		*C. ferox-2*	Shanxi, China
39	Taxa 16	*C. ferox* var. *thibetica-1*	Shanxi, China
40		*C. ferox* var. *thibetica-2*	Shanxi, China
41	Taxa 17	*C. wangii-1*	Yunnan, China
42		*C. wangii-2*	Yunnan, China
43	Taxa 18	*Ostryopsis davidiana-1*	Neimenggu, China
44		*Ostryopsis davidiana-2*	Neimenggu, China
45		*Ostryopsis davidiana-3*	Neimenggu, China

### High-Throughput Sequencing, Data Filtering, and SNP Genotyping

To generate genome-wide data to infer the evolutionary history of genus *Corylus*, we used Illumina sequencing of 2b-restriction site-associated DNA (2b-RAD). To guarantee the yield of DNA from herbarium material, genomic DNA was isolated using the modified cetyltrimethyl ammonium bromide (CTAB) protocol (Sobel and Streisfeld, 2015). The DNA concentration and purity were evaluated both with a NanoDrop-2000 spectrophotometer. The final concentration of DNA samples was diluted to 200 ng μL^-1^. 2b-RAD libraries were then prepared for each individual using the type IIB enzyme BsaXI, followed by single-end sequencing using an Illumina HiSeq X Ten platform, according to the protocol developed by Wang et al. (2012). We used the software Stacks v. 1.35 (Catchen et al., 2011) to remove reads with low quality or uncalled bases. Errors in the restriction site sequences and barcode were checked before downstream analysis. Then, reads were aligned to the finished reference genome from *Betula nana* (EMBL accession number ERP001867; Wang et al., 2013) using SOAP v. 2.21 (Li et al., 2009). SNP genotyping was performed using the program RADtyping v1.537 (Fu et al., 2013) with the maximum-likelihood (ML) algorithm (all remaining parameters as default). The resulted SNPs were further filtered for our subsequent analysis using the population module in Stacks v. 1.35, requiring that SNPs occur in at least 80% of the individuals and had a minimum minor allele frequency of 0.01 to exclude any SNP locus found in a single heterozygote.

### Phylogenetic Analysis

To infer relationships among samples, we performed phylogenetic analyses with concatenated SNP dataset using both the ML and Bayesian Inference (BI) methods. The optimal substitution models for the ML and BI phylogenetic analyses were determined by ModelFinder program (Kalyaanamoorthy et al., 2017) using the Bayesian information criterion (BIC), as implemented in IQ-TREE (Nguyen et al., 2014). The ML analysis was conducted with IQ-TREE using 1000 replicates of ultrafast bootstrapping (UFBoot: Minh et al., 2013) and 1000 bootstrap replicates of the Shimodaira/Hasegawa approximate likelihood-ratio test (SH-aLRT: Guindon et al., 2010). The BI analysis was performed in MrBayes 3.2 (Ronquist et al., 2012) by running for 100,000 generations and sampling every 100 generations with the selected evolutionary model. The run was not finished until the average standard deviation of split frequencies was lower than 0.01 in all cases. The first 25% of the trees were discarded as burn-in, and the remaining trees were used to construct a 50% majority-rule consensus tree and estimate the Bayesian posterior probabilities. Trees were visualized and edited in FigTree 1.4.0 (Rambaut, 2012).

### Recombination Tests and Phylogeny Reassessment

Recombination between nucleotide sequences is a major process influencing the evolution of most species. To track the potential recombination events between two introgressed ecotypes and their influence on phylogenetic inference, unguided tests for recombination were performed in the RDP4 program (Martin et al., 2015). RDP4 executes a hidden Markov model to estimate the breakpoint positions once a recombination event is identified. In our analysis, recombination tests were conducted with seven implemented algorithms using the RDP (Martin and Rybicki, 2000), GENECOV (Padidam et al., 1999), MaxChi (Smith, 1992), Chimaera (Posada and Crandall, 2001), BootScan (Martin et al., 2005), 3Seq (Boni et al., 2007), and SiScan (Gibbs et al., 2000) methods. We tested the SNP dataset alignment for recombination, with Bonferroni corrections applied to set the family-wise error rate to 0.05. Only results that were supported by at least four of the seven approaches were accepted.

Inference of phylogeny using genome-wide SNPs can be severely distorted by recombination events. Based on the recombination tests, we did detect three obvious recombination signals which may probably result in unreliable topologies. Therefore, we further conducted a modified phylogeny (ML and BI) using the recombination-free dataset to check the topological changes. The recombination-free dataset was generated from RDP4 and the method for phylogenetic analysis was the same as above.

### Admixture Analysis and Hybridization Detection

To test for admixture and to infer potential hybridization events between different clades, two Bayesian clustering procedures were applied. Firstly, the genetic structure was analyzed using software STRUCTURE 2.3.4 (Pritchard et al., 2000) to infer patterns of ancestry within *Corylus* species. Compared with phylogenetic methods, structure analysis reveals shared variation among inferred subgroups, which could result from admixture and hybridization. An admixture model with correlated allele frequencies was applied to identify the putative number of subgroups (*K*). Because the phylogenetic analysis revealed four major clades (Clades A–D) in the ingroup, we thus set the targeted *K* from 2 to 10, with 10 independent simulations for each *K* (100,000 burn-ins and 100,000 iterations). Then, Structure Harvester was used to capture the true number of populations as described by Earl (2012). Assignment to clusters was then compared to known clade composition in phylogenetic analysis.

Furthermore, the Bayesian model-based method implemented in NewHybrids v 1.1 (Anderson and Thompson, 2002) was further applied to compute the posterior probability that an individual belongs to distinct genotype frequency classes (two parents, F1 and F2 hybrids, and first generation hybrid backcrosses) corresponding to hybrid categories. We used 245 SNPs from our 2b-RAD dataset which were filtered the set of loci using the GenAlex 6.5 (Peakall and Smouse, 2012) to include SNPs with a minor allele frequency > 0.2. NewHybrids was executed with four replicate runs of 100,000 sweeps and a burn-in of 100,000 sweeps with default genotype categories. The Jeffrey’s priors were chosen to downweight the influence of an allele that might be rare in one species and absent in the other.

### Divergence Time Estimation

Molecular dating analysis was performed in BEAST 2.5 (Bouckaert et al., 2014), using an uncorrelated relaxed clock and a Yule speciation process to estimate the divergence time of *Corylus* at the interspecific level. This analysis involved 43 individuals representing distinctly separated genetic lineages or different species, of which *O. davidiana* was chosen as outgroup. For divergence time estimation, the general time-reversible nucleotide substitution model with among-site rate variation modeled with a gamma distribution (GTR + Γ) was selected. Based on previous biogeographical studies of order Fagales (Zhang, 2014), and two fossil records of *Corylus* species (Takhtajan, 1982; Wolfe and Wehr, 1987), three calibrations were selected in the analysis: (1) The root of the tree was calibrated with the stem age of *Ostryopsis*, using a normal distribution with a mean date of 37.2 Ma and a standard deviation of 4.0 (Zhang, 2014); (2) the stem age of *Corylus* was set to 41.7 Ma based on the fossil record (Wolfe and Wehr, 1987), using a normal distribution with standard deviation of 3.8 Ma; (3) the divergence date of the subsection *Colurnae* (Clade D) is based on the clear fossil record, which is a fossil fruit similar to modern *C. colurna* or *C. chinensis*, supporting the existence of this clade with a mean date 9.82 Ma (8.74–10.9 Ma) (Takhtajan, 1982). The input file for BEAST2, with all the parameters and priors, was set up in BEAUti 2.5 using the Bayesian method based on the Markov Chain Monte Carlo (MCMC) algorithm (Bouckaert et al., 2014). Molecular dating analysis was run for a total of 100 million generations with a sampling frequency of 1,000 generations. The adequacy of parameters was checked using Tracer v.1.6, noting effective sample size (ESS) values > 200. A 25% burn-in was applied in TreeAnnotator 2.1.2 (Rambaut and Drummond, 2014), and the posterior sample estimates of the trees were summarized and combined to produce a consensus maximum clade credibility tree. Finally, FigTree 1.4 (Rambaut, 2012) was used to visualize the best molecular phylogeny and the 95% highest posterior density (HPD) for each node.

### Ancestral Area Reconstruction

To reconstruct the broad-scale biogeographical history of *Corylus*, we coded the distribution of each extant species as a character with eight states according to floristic division proposed by Zhang et al. (2005) and Bassil et al. (2013): A, Northeast Asia; B, Qinling Mountains and Central Plains of China; C, Central and East China; D, southwestern China; E, the Himalayas; F, European-Mediterranean Area; G, eastern NA; H, western NA. Ancestral area reconstruction and the estimation of the spatial patterns of geographic diversification within *Corylus* were inferred using the Bayesian Binary MCMC (BBM) method, as implemented in RASP 4.0 (Yu et al., 2015). The input file for RASP came from the post burn-in trees from the interspecific BEAST analysis. Besides, a condensed tree used for mapping the ancestral distribution on each node was generated from TreeAnnotator. The BBM analysis was run under the fixed state frequencies model (Jukes–Cantor) with equal among-site rate variation for 2 million generations, 10 chains each, and 2 parallel runs. The number of maximum areas was maintained at four.

## Results

### SNP Genotyping

The sequencing of 45 2b-RAD libraries generated 255,951,619 raw reads (average 5,687,813 raw reads per sample). The average coverage of all genomes was 57.24×. After trimming the barcode, cleaning, and filtering out the low-quality reads, we obtained a total of 204,761,295 clean reads (average 4,550,251 clean reads), of which 181,770,561 were found to contain enzyme recognition sites (average 4,039,346 enzymes per sample). On average, the ratio of enzymes to clean reads in the sequencing libraries was over 74.8%, suggesting the high quality of the 45 libraries. Overall, an average of 60.40% of the high-quality reads for each sample was uniquely mapped onto the reference genome (Supplementary Table S1). Finally, a total of 4,894 SNPs were genotyped and used for subsequent analyses. The RAD data have been submitted to the Sequence Read Archive (SRA) database in the NCBI, under accession numbers SAMN09464508–SAMN09464552.

### Phylogenetic Analysis

**FIGURE 2:**
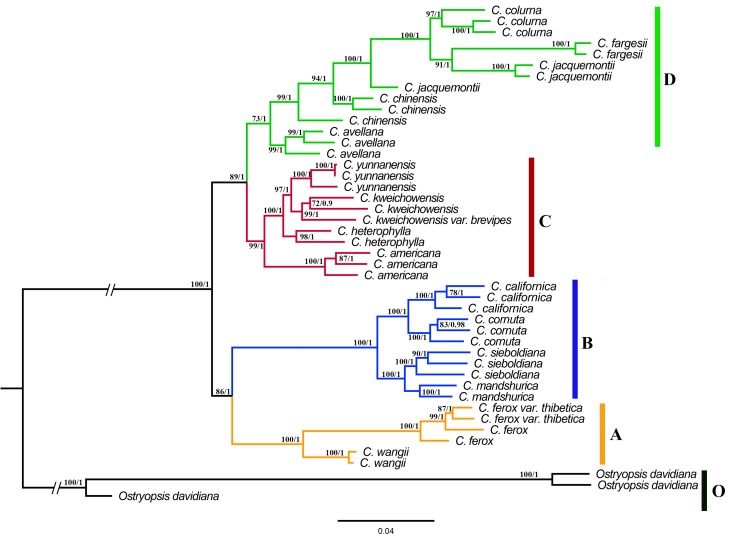
Maximum-likelihood (ML) tree of *Corylus* inferred from the concatenated SNP dataset. Four major clades designated in this study are highlighted with different color branches and vertical bars on the cladogram. Bootstrap values (BS) ≥ 70% in the ML analyses and posterior probabilities (PP) ≥ 0.95 in the Bayesian Inference (BI) analysis are listed above the branches (BS/PP). The hyphen refers to BS ≤ 70% in ML or PP ≤ 0.95 in BI.

Overall, phylogenetic trees inferred from ML and BI methods showed a highly consistent topology even at small branch nodes. Here, we only displayed the ML phylogeny inferred from TVMe + R2 model, with ultrafast bootstrapping (UFBoot > 70%) and Bayesian posterior probability (PP > 95%) values displayed above branches (Figure 2). Both phylogenetic trees identified four well-supported clades (A–D) in *Corylus* and resolved the phylogenetic relationships among the major clades. Clade A (UFboot/PP: 100/1) included three ancient *Corylus* species endemic to China: *C. ferox, C. ferox* var. thibetica, and *C. wangii*. *C. ferox* and its variety *C. ferox* var. thibetica clustered into a common subclade, with *C. wangii* formed its sister group. Clade B (100/1), a paraphyletic clade, was composed of four shrub species that disjunctively distributed between Northeast Asia and NA. In this clade, *C. cornuta* and *C. californica*, and *C. sieboldiana* and *C. mandshurica* formed two separate subclades, respectively. Clade C (99/1) was a species complex consisted of five morphologically similar shrubs: *C.* *americana, C. heterophylla, C. yunnanensis, C. kweichowensis*, and its variety *C. kweichowensis* var. brevipes. Of these species, four Chinese species (*C. heterophylla, C. yunnanensis, C. kweichowensis*, and its variety) showed a closer affinity than that with *C. americana* of American origin. Clade D (73/1) was a multi-origin group of five geographically isolated species, including *C. colurna* and *C. avellana* from European-Mediterranean region, *C. jacquemontii* from the Himalayas, and *C. fargesii* and *C. chinensis* from China. Notably, this clade was moderately supported by ultrafast bootstrap approximation.

### Recombination Tests and Phylogeny Reassessment

**FIGURE 3:**
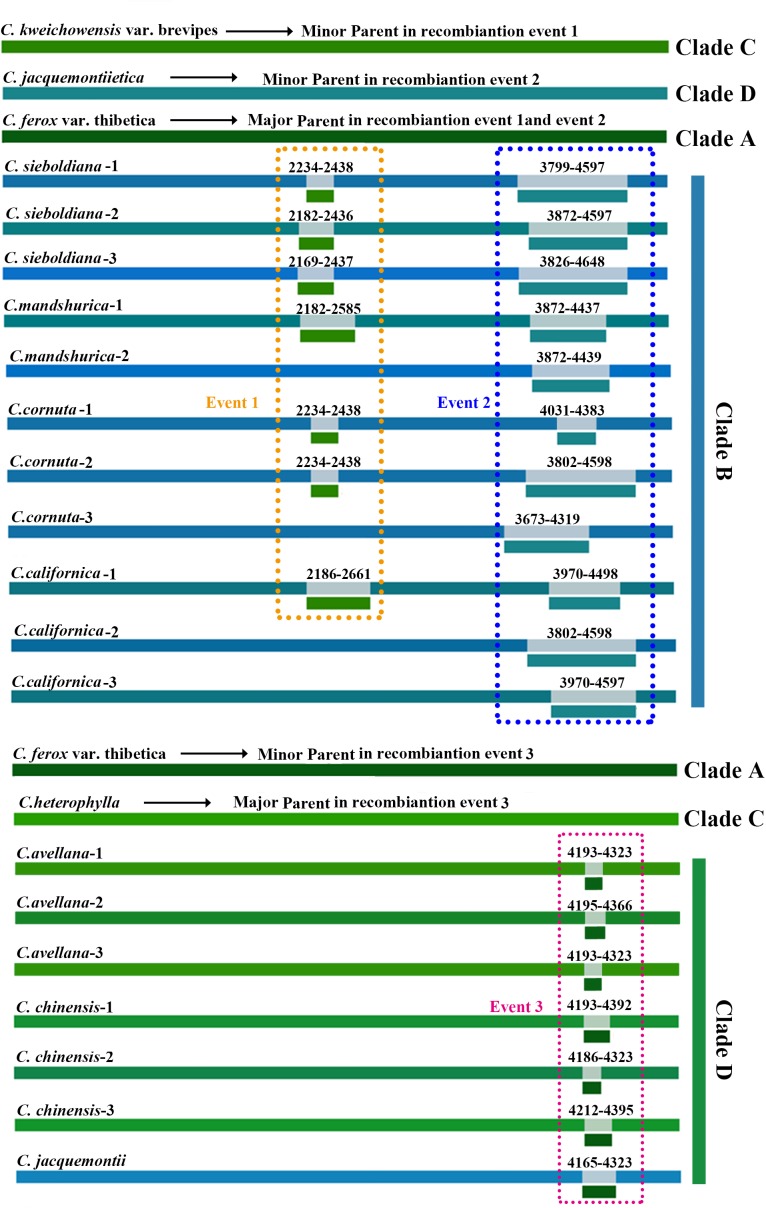
Recombination events predicted using the RDP4 program. Three recombination events (Event 1–Event 3) supported by at least four methods are displayed. The putative recombinant segments associated with each recombination event are indicated in colors. The detailed positions of all recombination break points are also listed.

Three putative recombination events were identified by at least four of the seven methods in RDP4 (Table 2). Both the first two recombination events were discovered to occur in Clade B (Figures 1, 2), with all the individuals involved as recombinants. The potential major and minor parents in the first recombination event were appointed to *C.* *kweichowensis* var. brevipes in Clade C and *C. ferox* var. thibetica in Clade A. ML breakpoints of these recombinants were roughly located within the 2,100–2,500 nucleotide regions (Figure 3). The second recombination event was highly supported by six of the seven methods (Table 2). An approximately 700 bp recombinant segments originated from the major parent *ferox* var. thibetica (Clade A) and the minor parent *C. jacquemontii* (Clade D), a species distributed in the Himalayas. The breakpoint of each recombinant was displayed to be seated within the 3,800–4,300 nucleotide regions. A third recombination event was found to occur in Clade D (Figures 1, 2), with three of the five species (*C. chinensis, C. avellana*, and *C. jacquemontii*) being related to recombinants (Table 2 and Figure 3). A small recombinant segments (∼150 bp) may have originated from two unknown parents similar to *C. ferox* var. thibetica (Clade A) and *C. heterophylla* (Clade C), respectively.

**Table 2:** Bonferroni corrected *P*-values for the three recombination events detected in Clades B and D.

Recombination	Clade B-event 1	Clade B-event 2	Clade D-event 3
tests	(*p*-value)	(*p*-value)	(*p*-value)
(methods)			
RDP	2.10 × 10^-01^	2.89 × 10^-01^	5.06 × 10^-03^
GENECOV	–	–	–
BootScan	1.11 × 10^-04^	2.72 × 10^-03^	1.94 × 10^-02^
MaxChi	5.38 × 10^-03^	1.73 × 10^-02^	–
Chimaera	3.41 × 10^-02^	8.16 × 10^-03^	4.76 × 10^-02^
SiScan	1.08 × 10^-02^	6.75 × 10^-04^	–
3Seq		1.39 × 10^-02^	2.03 × 10^-02^

**FIGURE 4:**
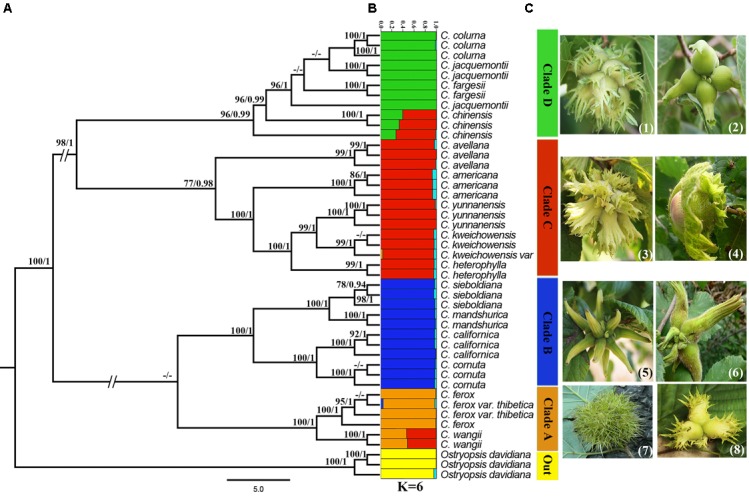
Phylogeny reassessment and admixture analysis. **(A)** Phylogeny reassessment using the recombination-free dataset. Bootstrap values (BS) ≥ 70% in the ML analyses and posterior probabilities (PP) ≥ 0.95 in the BI analysis are listed above the branches (BS/PP). The hyphen refers to BS ≤ 70% in ML or PP ≤ 0.95 in BI. **(B)** Genetic structure of *Corylus* species through an admixture analysis implemented in STRUCTURE. The *x*-axis showed different individuals and species, with numbers in parentheses representing taxa and numbers outside the parentheses representing single sample, respectively. The *y*-axis quantified the membership probability of accessions belonging to different clusters. Colors in each row represented structural components. **(C)** The phenotypic traits of husks of eight representative species in each clade, with (1)–(8) correspond *C. colurna, C. chinensis, C. americana, C. heterophylla, C. sieboldiana, C. mandshurica, C.* *ferox*, and *C. wangii*, respectively.

In order to assess the influences of recombination signals on tree topology, an additional phylogenetic analysis was conducted using the recombination-free data (Figure 4A). Compared to prior phylogenetic tree (Figure 2), the newly generated tree showed a marked change by transferring *C. avellana* from Clade D to Clade C. This change was closely related to these recombination events, especially the third one, from which *C. avellana* may have obtained recombinant fragments shared by species in Clade D. However, the species composition and topology of other clades remained unchanged.

### Admixture Analysis and Hybridization Detection

All individuals were further assessed for genetic stratification using the STRUCTURE program. SNP data were analyzed with the possible clustering number (*K*) ranging from 2 to 10. The Δ*K* showed a clear maximum for *K* = 6 (Supplementary Figure S1 and Figure 1), indicating that all individuals (including outgroup) could be classified into six optimal subgroups. From the structure plot (Figure 4B), we identified five clear subgroups which corresponded consistently with five clades (A–D and outgroup) of the recombination-free phylogeny (Figure 4A). Simultaneously, we also captured an inconspicuous but extensively existed subgroup, suggesting a complex pattern of introgression and admixture among different clades. Particularly, *C. chinensis* and *C. wangii* seem to have originated from the hybridization between Clades C and D, and Clades C and A, respectively (Figures 4A,B).

Using the subset of 245 SNPs with a minor allele frequency *>* 0.2, genotype frequency classes of each individual were calculated by NewHybrids (Supplementary Table S2 and Table 2). With a probability of 1, all the 11 individuals in Clade B designated by STRUCTURE were assigned as the pure parent 1, while the 25 individuals in Clade C + Clade D were classified as another pure parent 2. Interestingly, NewHybrids identified no individuals to be F1 hybrids, while four individuals of *C. ferox* and *C. ferox* var. thibetica in Clade A were tagged as F2 with a probability of 1. Furthermore, two individuals of *C. wangii* were classified by NewHybrids as backcross hybrids (F2 × pure parent 2). It is noteworthy that the majority of these hybrids are from the same Clade A, indicating that the species in this clade are probably more easily to hybrid with species of other clades.

### Divergence Time Estimation

**FIGURE 5:**
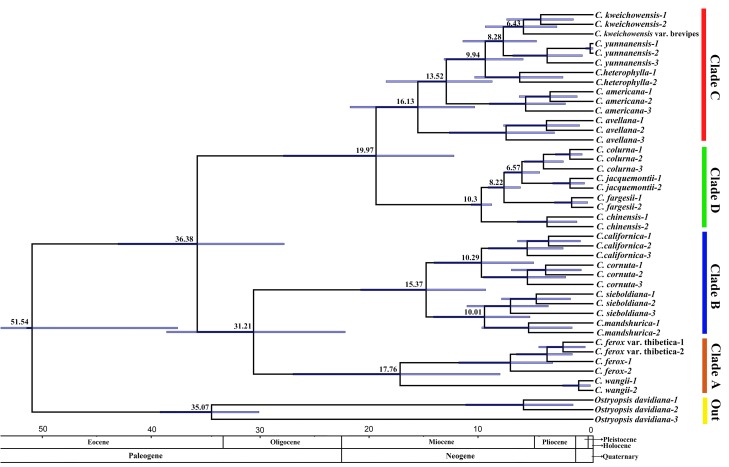
Phylogenetic chronogram showing the divergence times estimated in BEAST. The divergence times of the clades and subclades are shown near each node. Blue bars represent 95% HPD for the estimated mean dates. The clades (A–D) correspond to those in Figure 4A.

The tree topology recovered from the molecular dating analysis (Figure 5) was identical to those inferred from the ML analysis and BI using the recombination-free data (Figure 4A). All the nodes in the tree were highly supported with a posterior probability of >0.99 (Figure 5). The age of the most recent common ancestor (MRCA) of *Corylus* and *Ostryopsis* was estimated by the BEAST analysis to be 51.54 Ma, with the 95% HPD ranging from 38.18 to 66.62 Ma. The MRCA of *Corylus* species began to occur in late Eocene (36.38 Ma, 95% HPD: 28.39–43.65 Ma), which was slightly earlier than the divergence time of *Ostryopsis* (35.07 Ma, 95% HPD: 30.71–39.79 Ma). Within the genus *Corylus*, two major clades started to diverge in different directions at around 31.21 (95% HPD: 22.79–39.19 Ma) and 19.97 Ma (95% HPD: 12.81–28.47 Ma), respectively. In the early and middle Miocene (10.3–17.76 Ma), the rudiments of four modern clades have basically taken shape. Subsequently, rapid speciation in different clades occurred in the middle and late Miocene. The divergence between *C. wangii* and the section *Acanthochlamys* (*C. ferox* and *C. ferox* var. thibetica) in Clade A was estimated to be 17.76 Ma (95% HPD: 8.58–27.6 Ma). In Clade B, the intercontinental division between Asian species (*C. sieboldiana* and *C. mandshurica*) and American species (*C. cornuta* and *C. californica*) took place about 15.37 Ma (95% HPD: 9.9–21.38 Ma), while the split time within each subclade was identically around 10 Ma. The species differentiation in Clade C has experienced an incremental process, of which *C. avellana* and *C.* *americana* separated successively at about 16.13 and 13.52 Ma, while the *C. heterophylla* complex (*C. heterophylla, C. yunnanensis, C. kweichowensis*, and its variety) showed a rapid speciation (6.43–9.94 Ma). *C. chinensis* was the first to separate from Clade D 10.3 Ma ago, whereas the divergence between *C. jacquemontii* and *C. colurna* was estimated to be 6.57 Ma.

### Ancestral Area Reconstruction

**FIGURE 6:**
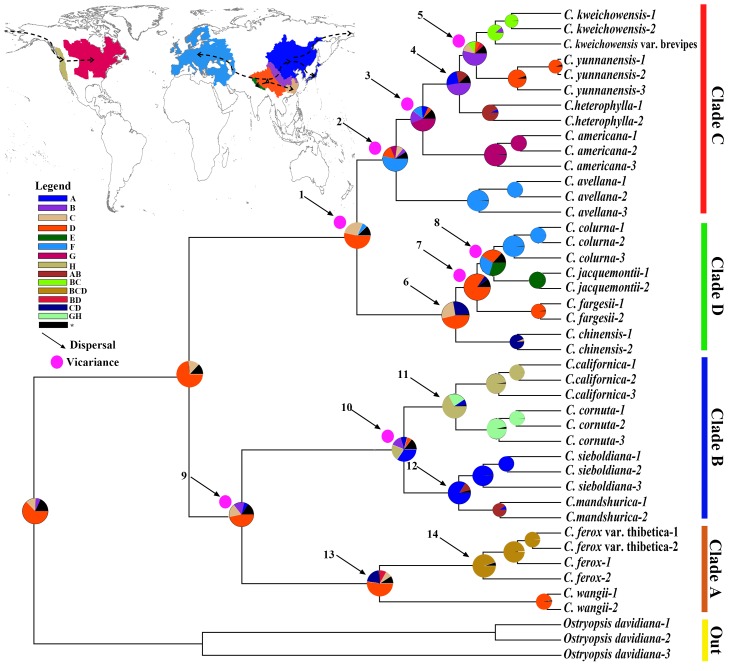
Ancestral area reconstructions based on the BBM method in RASP. The insert map shows the geographical distribution of *Corylus* species, overlaid on major floristic divisions (A–H) according to Zhang et al. (2005) and Bassil et al. (2013). Letters and colors in the legend refer to extant and possible ancestral areas, and combinations of these. Inferred dispersal and vicariance events are indicated by black arrows and red pies, respectively. Pie charts on each node indicate marginal probabilities for each alternative ancestral area, with the maximum area number set to four.

The results of the ancestral area reconstruction were shown in Figure 6. BBM reconstruction indicated that southwestern China (D) was the most probable ancestral area for ancient *Corylus* species. Furthermore, results of the BBM analysis suggest that dispersal and vicariance played a substantial role in the biogeographic history of *Corylus*. Four long distance dispersal (LDD) events (nodes 1, 3, 4, and 5) from southwestern China to NA (route: D–C–B–A–G) and subsequent vicariance events (nodes 1, 3, and 5) were derived, forming four distinct lineages in Clade C: *C.* *americana, C. heterophylla, C. yunnanensis, C. kweichowensis*, and its variety *C. kweichowensis* var. brevipes. Almost the same route across the BLB (D–C–B–A–H), Clade B became an independent lineage through four dispersal (nodes 9, 10, 11, and 12) and two vicariance events (nodes 9 and 10). Furthermore, another two independent dispersal routes passing from southwestern China to the Himalayas (route: D–E; node 2) and European-Mediterranean area (route: D–F; node 8) were also predicted. Along with subsequent vicariance events (nodes 2 and 8), *C. avellana* in Clade C and *C. jacquemontii* and *C. colurna* in Clade D became distinct species gradually. Besides, sympatric and parapatric speciation patterns with short distance dispersals were observed, including *C. wangii* and *C. ferox* in Clade A (nodes 13 and 14), and *C. chinensis* and *C. fargesii* in Clade D (nodes 6 and 7).

## Discussion

### Speciation Mechanisms and Rapid Diversification

Results of the above analyses revealed strong recombination (Table 2 and Figure 3) and hybridization signals (Figure 4B; Supplementary Table S2), as well as clear dispersal and vicariance events (Figures 5, 6), which have more or less influenced the speciation and diversification process of the genus *Corylus*. Phylogenies are always constructed assuming that nucleotide sequences replicate without recombining. However, it is very likely that recombination can severely bias population and phylogenetic analyses and finally lead to incorrect results (Martin et al., 2017; Kiil and Østerlund, 2018; Schierup and Hein, 2000). SNP nucleotide sequences of nuclear genome have the characteristics of biparental inheritance, which potentially contain recombination events. Accordingly, inference of phylogeny using genome-wide SNPs can be severely distorted by recombination events either between sequences within the dataset or with an unobserved sequence. In this study, we discovered plenty of recombination events and simultaneously verified their influences on phylogeny. The fact indicates that all the four clades (A–D) have involved in recombination either as potential recombinants or parents (Figures 2, 3), of which almost all the taxa in Clade B and partial taxa in Clade D belong to recombinants while individuals from Clade A, Clade B, and Clade C are identified as recombinant parents. This universal phenomenon of interspecific recombination among *Corylus* species demonstrates the existence of hybridization, during which process the recombinants obtain the genetic components of both parents. The divergence of the two phylogenies based on concatenated and free-recombination dataset centers on the phylogenetic position of *C. avellana* which is classified into Clade C by the free-recombination dataset (Figure 4A) but ranked into Clade D by concatenated dataset (Figure 2). It turns out that the free-recombination phylogeny shows high consistency with previous morphological classification and ITS phylogeny (Erdogan and Mehlenbacher, 2000; Whitcher and Wen, 2001). Structure analysis also reveals extensive introgression existing in different clades, especially evident between Clades C and A, and Clades C and D (Figure 4B). Similarly, hybridization signals are also predicted by NewHybrids detection (Supplementary Table S2). All the above analyses demonstrate that recombination and hybridization are important mechanisms for the speciation of *Corylus* species.

Dispersal–vicariance model is a significant mechanism in speciation, especially for the LDD. Divergence time estimation and ancestral area reconstruction indicate that the ancestors of *Corylus* originate from the southwestern China (D) in late Eocene (∼36.38 Ma) (Figures 5, 6). Southwestern China, a region originated from the Qinghai–Tibetan Plateau (QTP) uplift, is finally shaped as one of the biodiversity hotspot in the north temperate region and is regarded as the potential original center of many modern species (Li and Li, 1993; Wen et al., 2014; Deng et al., 2017). Thus, it is no wonder that *Corylus* has also originated from this diversity center. However, subsequent dispersal displays diverse patterns according to their dispersal routes. One of the most important routes involves the LDD from southwestern China to northeast Asia (D–C–B–A) and then across the BLB to NA (G/H), through which Asian bristle-husked shrubs (*C. sieboldiana* and *C. mandshurica*) in Clade B and leaf-husked shrub (*C. heterophylla*) in Clade C dispersed to NA at a very close time (∼15.37 and 13.52 Ma). Rapid species diversification in the two clades occurred approximately between 6 and 16 Ma, during which time geological isolation after dispersal may have played an important role. Besides, the ancestor of leafy husked shrubs is found to pass through West Asia to the European-Mediterranean area, which is slightly earlier (0.76 Ma) than the BLB dispersal, forming the famous European species *C. avellana* (Clade C). The modern species of Clade D are related with three speciation patterns: allopatric (*C. colurna* and *C. jacquemontii*), sympatric (*C. fargesii*), and parapatric (*C. chinensis*) speciation. The speciation time of Clade D falls into the late Miocene (6–10 Ma), which is fairly close to the divergence of several linkages in Clade C and is probably caused by the global geologic and climatic changes in East Asia during that time. Particularly, the rise of the Himalayas is probably the major cause to bring about the divergence between *C. colurna* and *C. jacquemontii*. Clade A differentiated firstly from *Corylus* at about 17.76 Ma, suggesting a more ancient origin than other clades. Notably, this clade involves extensive hybridization and recombination events, which are likely to be the genetic residual of ancient hybridization.

### Evolutionary Relationships and Taxonomic Implications

Relationships among *Corylus* species have been studied previously either through phenotypic characters (Ferreira et al., 2010; Ciarmiello et al., 2014) or molecular markers (Erdogan and Mehlenbacher, 2000; Boccacci and Botta, 2010; Martins et al., 2014; Mohammadzedeh et al., 2014). However, none of them have reached definitive conclusions for the phylogenetic relationships and taxonomy of *Corylus* species partly due to incomplete taxa sampling by lacking some rare species such as *C. ferox, C. fargesii, C. wangii*, and *C. chinensis*, and partly because of the low resolution of molecular markers including SSR marker, *mat*K gene, and ITS regions. In our study, detailed sampling was conducted by collecting almost all the *Corylus* species generally accepted in the current, and integrated analyses were performed by combining phylogenetics and structure inference. Although ML and BI phylogenies revealed identical classification results by dividing the ingroup into four clades (Figure 2), it is confirmed that the phylogenetic topology has been influenced by recombination events (Figure 3), which finally resulted in the misjudgment on the phylogenetic position of *C. avellana* (Figure 2). However, a robust phylogeny is successfully reconstructed based on the integrated inference from structure analysis and recombination tests (Figure 4A). That is, four distinct clades (A–D) are identified by transferring *C. avellana* from Clade D to Clade C. In the following, we will discuss each of these clades, respectively.

### Clade A

Clade A is comprised of three ancient species (variety): *C. wangii, C. ferox*, and its variety *C. ferox* var. thibetica. *C. ferox* was once suggested to be the basalmost extant taxon of the genus based on morphological traits and ITS phylogeny (Li and Cheng, 1979; Whitcher and Wen, 2001). It is very distinct from other *Corylus* species in fruit involucre in that it has bur-like spiny husks that resemble those of chestnuts (Figure 4C). Despite that *C. ferox* and *C. ferox* var. thibetica are not well separated by molecular phylogeny in this study, potential phenotypic differentiation can still provide valid evidence to distinguish them. The visible difference lies in their leaf size and shape, with leaves of the variety being more spacious than native species (Figures 1L,M). *C. wangii* has been seldomly studied by researchers due to its limited geographical distribution. However, it is definitely a unique species that segregates from other *Corylus* species but displayed a close affiliation with *C. ferox*. Integrated analyses of structure analysis (Figure 4B) and hybridization detection (Supplementary Table S2) indicate that *C. wangii* is probably originated from ancient hybridization between Clades A and C. Molecular dating analysis supports the earliest differentiation of Clade A (Figure 5), revealing an ancient origin as described above. However, the resulted phylogeny does not reflect the monophyletic or basal position of this clade but assign it as the sister to Clade B. The discordance may probably be caused by incomplete sampling for *Corylus* species or the low resolution of markers in previous studies.

### Clade B

Clade B is the most robust paraphyletic group that consists of two North American species *C. californica* and *C. cornuta*, and two East Asian species *C. sieboldiana* and *C. mandshurica*. This clade in our results is in agreement with those of Erdogan and Mehlenbacher (2000), Whitcher and Wen (2001), and Bassil et al. (2013). The remarkable features of these species are their tubular and beaked husks with very loosely attached bristles (Figures 1Q,X, 4C). Although these species exhibit disjunctive distribution between East Asia and NA, multivariate data from interspecific hybridization relations, phenotypic characters, and molecular markers reveal high similarities and close affinity among these species. Distribution of *C. mandshurica* displays apparent dispersal from southwest to northeast in China, while *C. sieboldiana* mainly distributes in the adjacent regions such as Korean peninsula and Japanese archipelago. Thompson et al. (1996) proposed *C. mandshurica* was synonyms or variety of *C. sieboldiana*, and Chin et al. (2004) noted that the two belonged to different populations of the same species. In view of their separate distribution areas and the inferred dispersal route, it is obvious that *C. mandshurica* is the original species, while *C. sieboldiana* is a derivative. Therefore, we do not support the designation that *C. mandshurica* was the variety of *C. sieboldiana*. On the contrary, we suggest *C. sieboldiana* to be a variety of *C. mandshurica* or a distinct species. *C. californica* and *C. cornuta* distribute in two different regions of NA: western NA (H) and eastern NA (G), respectively. The taxonomic status of *C. californica* has long been controversial. It was once viewed as a distinct species by some researchers (Krüssmann, 1976; Erdogan and Mehlenbacher, 2000), but as a botanical variety (Everett, 1982; Thompson et al., 1996; Huxley and Griffiths, 1999) or a subspecies (Furlow, 1997) of *C. cornuta* by others. In the present study, *C. californica* and *C. cornuta* formed their separate group in the phylogeny (Figure 4A), suggesting the genetic divergence between them. Notably, ancestral area reconstruction reveals a dispersal route from western NA (H) to eastern NA (G), which probably means that the occurrence of *C. cornuta* was later than *californica.* Moreover, the easy cross between the two may support the botanical variety designation. Similarly, we would put forward a different opinion that *C. californica* is the product of LDD crossing BLB of Asian *C. mandshurica* and *C. sieboldiana*, while *C. cornuta* is a geographical variety of *C. californica* or have become a distinct species. The deep-rooted relationships in this clade remain to be further investigated through population genetics.

### Clade C

Clade C is formed by six leafy-husked shrubs that disjunctively distribute among East Asia, Europe, and NA, of which, four Chinese species (*C. heterophylla, C. yunnanensis, C. kweichowensis*, and its variety *C. kweichowensis* var. brevipes.) constitute the *C. heterophylla* complex, while the other two species (*C. americana* and *C. avellana*) form their separate subclade. It is interesting that these species group together although they are geographically isolated. The potential relationships among them can be described using the morphological similarity especially for husks and nuts. The typical husk characters of these species are leafy-shape, with deep incisions in the margin and no constriction at the tip (Figures 1P, 4C). Furthermore, it is verified that the three species *C. americana, C. avellana*, and *C. heterophylla* can hybridize easily with each other under natural conditions, with the hybrid variety “*C. heterophylla* × *C. avellana*” as a typical case. Divergence time estimation and ancestral area reconstruction indicate that *C. avellana* and *C. americana* successively spread to Europe and NA via two different dispersal routes in early and middle Miocene, respectively. Species differentiation in the *C. heterophylla* complex is found to occur recently, which may lead to the difficulty in species identification. Once, *C. kweichowensis* and *C. yunnanensis* were both viewed as botanical varieties of *C. heterophylla* by some researchers (Yu, 1979; Thompson et al., 1996) and as distinct species by others (Liang and Zhang, 1988; Qi, 1996; Ma et al., 2014). Besides, *C. kweichowensis* var. brevipes, a variety with typically brachypodous characteristics was also identified (Liang and Zhang, 1988). In our study, the phylogenies support the close affinity among *C. heterophylla, C. kweichowensis*, and *C. yunnanensis* (Figure 4A). *C. heterophylla* and *C. yunnanensis* that locate at two terminals of the dispersal route differentiate earlier than *C. kweichowensis* in the middle zone. We infer that the significant differences in ecological conditions between south and north of China as well as the frequent gene flow in the middle zone have affected the species differentiation in this complex. Remarkably, a vicariance event (node 5 in Figure 6) occurred in the late Miocene between *C. kweichowensis* and *C. yunnanensis*, providing more support for the designation of distinct species.

### Clade D

Clade D is a well-resolved phylogenetic group composed by four tree species: *C. chinensis, C. colurna, C. jacquemontii*, and *C. fargesii*. The clustering group of *C. chinensis, C. colurna*, and *C. jacquemontii* was previously demonstrated by nuclear microsatellite-based clustering (Bassil et al., 2013), strict consensus tree of *mat*K gene, and ITS regions (Erdogan and Mehlenbacher, 2000; Whitcher and Wen, 2001). Although there are few reports about the molecular taxonomy of *C. fargesii* in previous studies, both the phylogenetic and structure analysis classify this species into the Clade D. Therefore, we support Clade D as a reliable clustering group. The common phenotypic characters of these four species are their big single stems and juicy-flesh husks covered with glandular hairs (Figures 1C–F, 4C). Both *C. chinensis* and *C. fargesii* own the constricted tubular husks that prevent the nuts falling even after maturity (Figures 1R,S). *C. fargesii*, also called the paperbark tree hazel, is native to China and morphologically distinct from other tree species, with its bark exfoliating like *Betula* species (Figure 1Y). *C. colurna* from the Mediterranean regions is recognized as the carbon copy of *C. jacquemontii* that distributes in the Himalayas by presenting not only extremely similar growth habit, but also analogous husk and nut morphology. We infer that the divergence between the two is caused by the rise of the Himalayas.

## Author Contributions

T-TZ and G-XW conceived and designed the experiments. ZY, T-TZ, G-XW, Q-HM, and L-SL participated in the collection of study materials. ZY and T-TZ participated in the DNA extraction and data analysis. ZY wrote the manuscript. All authors read and approved the final manuscript.

## Conflict of Interest Statement

The authors declare that the research was conducted in the absence of any commercial or financial relationships that could be construed as a potential conflict of interest.

## Footnotes

1 http://www.theplantlist.org

## Supplementary Material

FIGURE S1 The log probability of the Δ*K*-value for 10 replicated STRUCTURE runs given each number of clusters *K* based on genome-wide SNPs.

TABLE S1 Summary of high-throughput sequencing.

TABLE S2 The estimated posterior probabilities generated with NewHybrids that each individual belongs to each of the different genotype frequency categories.
