# Supplementary figures and images for: Resolving the Speciation Patterns and Evolutionary History of the Intercontinental Disjunct Genus Corylus (Betulaceae) Using Genome-Wide SNPs
Source: Front Plant Sci. 2018 Oct 25;9:1386. doi: 10.3389/fpls.2018.01386 (PMC6209643; doi:10.3389/fpls.2018.01386)

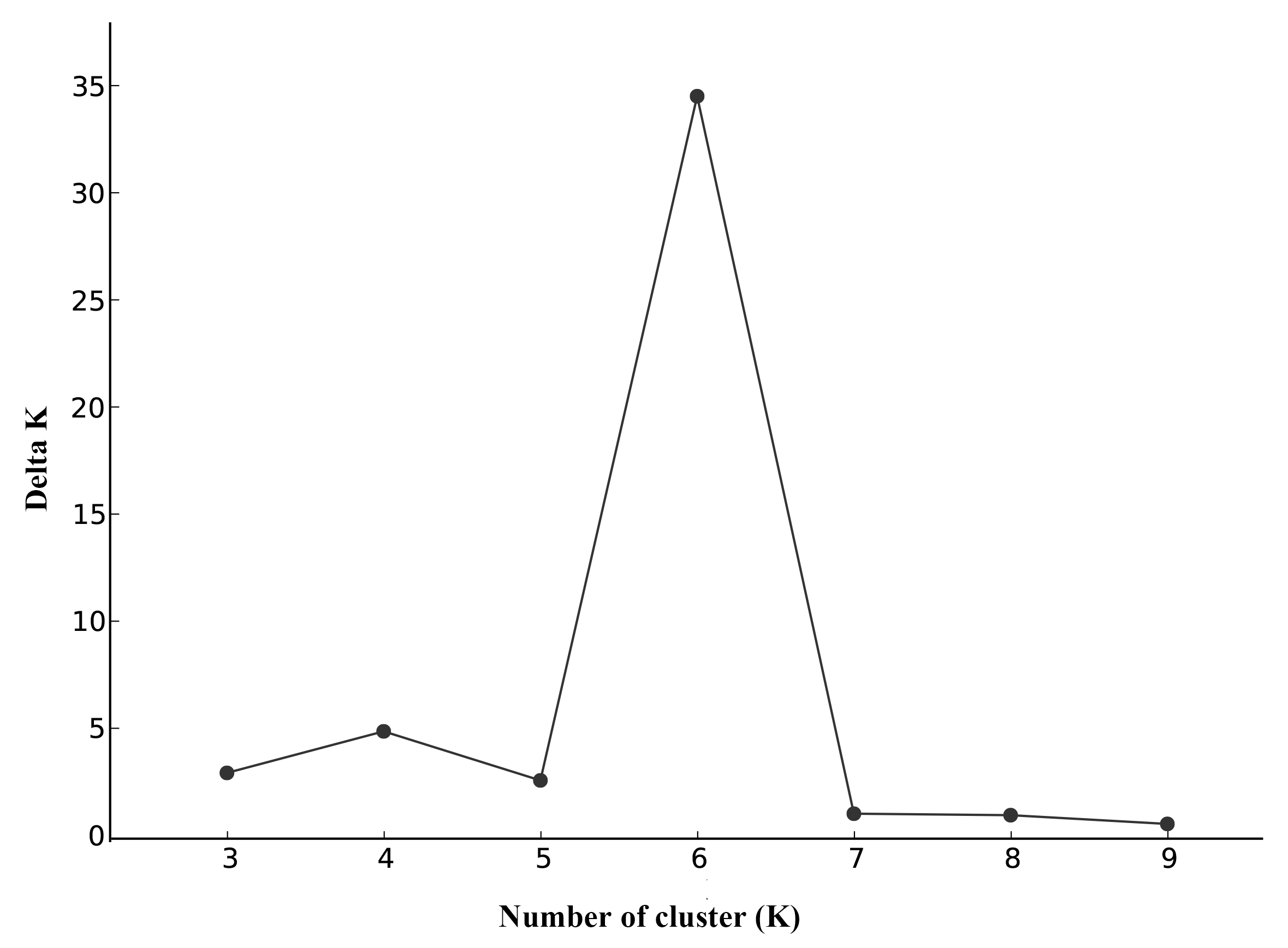

Supplement: FIGURE S1 — The log probability of the ΔK-value for 10 replicated STRUCTURE runs given each number of clusters K based on genome-wide SNPs. [file Image_1.TIF]
